# Poor speech recognition, sound localization and reorganization of brain activity in children with unilateral microtia-atresia

**DOI:** 10.1007/s11682-021-00478-9

**Published:** 2021-07-10

**Authors:** Qiang Liu, Yibei Wang, Tengyu Yang, Yue Fan, Bo Hou, Yushan Chen, Jian Wang, Xiaowei Chen

**Affiliations:** 1 Department of Otolaryngology, Peking Union Medical College Hospital, Beijing, 100730 China; 2 Department of Otolaryngology, Beijing TianTan Hospital, Capital Medical University, Beijing, 100050 China; 3 Department of Otolaryngology, China-Japan Friendship Hospital, Beijing, 100029 China; 4 Department of Radiology, Peking Union Medical College Hospital, Beijing, 100730 China; 5 The Ohio State University, Columbus, OH 43201 USA; 6 School of Communication Science & Disorders, Dalhousie University, 5850 College Street, Halifax, B3H1X5 Canada

**Keywords:** Unilateral microtia-atresia, Speech recognition, Sound localization, Cognitive function, Rs-fMRI, Brain networks

## Abstract

Microtia-atresia is a congenital malformation of the external ear, often affecting one side and being associated with severe-to-profound unilateral conductive hearing loss (UCHL). Although the impact of unilateral hearing loss (UHL) on speech recognition, sound localization and brain plasticity has been intensively investigated, less is known about the subjects with unilateral microtia-atresia (UMA). Considering these UMA subjects have hearing loss from birth, we hypothesize it has a great effect on brain organization. A questionnaire on speech recognition and spatial listening ability was administered to 40 subjects with UMA and 40 age- and sex-matched controls. UMA subjects showed poorer speech recognition in laboratory and poorer spatial listening ability. However, cognitive scores determined by the Montreal Cognitive Assessment (MoCA) and Wechsler Intelligence Scale for Children (WISC-IV) did not differ significantly in these two groups. The impact of hearing loss in UMA on brain functional organization was examined by comparing resting-state fMRIs (rs-fMRI) in 27 subjects with right-sided UMA and 27 matched controls. UMA subjects had increased nodal betweenness in visual networks and DMN but decreases in auditory and attention networks. These results indicate that UCHL in UMA causes significant abnormalities in brain organization. The impact of UCHL on cognition should be further examined with a battery of tests that are more challenging and better focused on the cognitive networks identified.

## Introduction

Congenital microtia-atresia manifests as developmental defects of the external ear and, in many subjects, of the middle ear (Luquetti et al., 2011). The incidence of microtia-atresia in China has been estimated at 0.81–1.53 per 10,000 live births, with approximately 90% of affected individuals having unilateral microtia-atresia (Deng et al., 2016). Patients with congenital microtia-atresia often have severe conductive hearing loss on the affected side, resulting in attenuated transmission of acoustic signals to the cochlea via air conduction. Although previous studies have reported deficits in speech perception and sound localization in patients with unilateral sensorineural hearing loss (USHL) (Asp & Reinfeldt, 2019; Bess & Tharpe, 1988; Bess et al., 1986; Schmithorst et al., 2014), few have focused on the impact of unilateral conductive hearing loss (UCHL) on these functions in children, with none of these studies evaluating the effects of UCHL on hearing function in subjects with congenital unilateral microtia-atresia (UMA).

Speech and language skills were thought to develop normally without major sequelae in children with UMA, with hearing amplification considered unnecessary. However, unilateral hearing loss (UHL), including conductive loss, has been shown to have a negative impact on speech recognition and directional discrimination of sound in noisy environments by children (Asp et al., 2018; Griffin et al., 2019), as well as to negatively affect children’s cognition and academic performance (Anne et al., 2017; Lieu., 2018; Rohlfs et al., 2017; van Hovell Tot Westerflier, 2018). Specifically, poorer academic performance was reported in two studies of children with UMA (Jensen et al., 2013; Reed et al., 2016), although the data might have been biased by the study design (van Hovell Tot Westerflier et al., 2018). Therefore, the impact of UCHL on hearing, cognitive function and brain organization in children with UMA should be thoroughly investigated and the need for early hearing intervention in these children should be verified.

Resting-state functional magnetic resonance imaging (rs-fMRI) is a noninvasive technique that measures low-frequency fluctuations of blood oxygen level-dependent (BOLD) signals at rest. These fluctuations reflect spontaneous neural activity of the brain (Li et al., 2018; Smitha et al., 2017; Zhang et al., 2019). Abnormal brain activities associated with executive function, cognition and language comprehension have been found in children with severe-to-profound USHL (Jung et al., 2017; Tibbetts et al., 2011). However, little is known about the effects on these functions of severe UCHL occurring before birth. Because hearing loss (HL) in these subjects is established before the critical period of brain development, the impact of this HL on cognition and other brain functions may be more significant than that of HL that occurs after this critical period.

This study compared speech recognition, sound localization, cognitive abilities and spontaneous brain activities in children with UMA with those of age/gender matched controls. Because the impact on academic performance was not as profound in children with UMA as in children with USHL (Kesser et al., 2013), this study evaluated whether the changes in resting-state brain activities observed in subjects with USHL were also present in subjects with UCHL due to UMA. The outcomes of this study support the need for early hearing rehabilitation in children with UMA.

## Materials and methods

### Subjects and procedures

This study involved 40 children with UMA (27 right-sided and 13 left-sided) and 40 age- and sex-matched normal controls. All participants were right-handed. The subjects in the UMA group presented with severe UCHL. All subjects were raised in monolingual Mandarin-speaking families with normal-hearing parents, and all were intellectually and neurologically normal. None of the subjects received hearing aids before this study.

During their first visit upon recruitment, all subjects filled out a consent form and completed a questionnaire (with the help of their parents if necessary) addressing sound localization ability with the focus on speech signal. The speech recognition and spatial localization abilities of the subjects were examined, in addition to regular pure tone audiometry tests. Subsequently, all subjects completed cognition tests, including the Montreal Cognitive Assessment (MoCA) and Wechsler Intelligence Scale for Children (WISC-IV). Most subjects underwent rs-fMRI examinations on a separate day (visit). To avoid any potential effects resulting from the side of deafness, only the 27 children with right-sided microtia-atresia and the same number of control subjects underwent rs-fMRI examinations. Subjects with contraindications to MRI were excluded. The study protocol was approved by the Institutional Ethical Review Board of Peking Union Medical College Hospital.

### Hearing evaluation

All audiological tests were performed by qualified medical assistants in a soundproof room. Pure-tone average (PTA) thresholds across the conventional frequency range of hearing tests (0.25, 0.5, 1, 2, 4, and 8 kHz) were determined using a manual audiometer (GSI-61, Grason-Stadler Inc, Denmark) coupled with TDH-39 headphones.

**Fig. 1:**
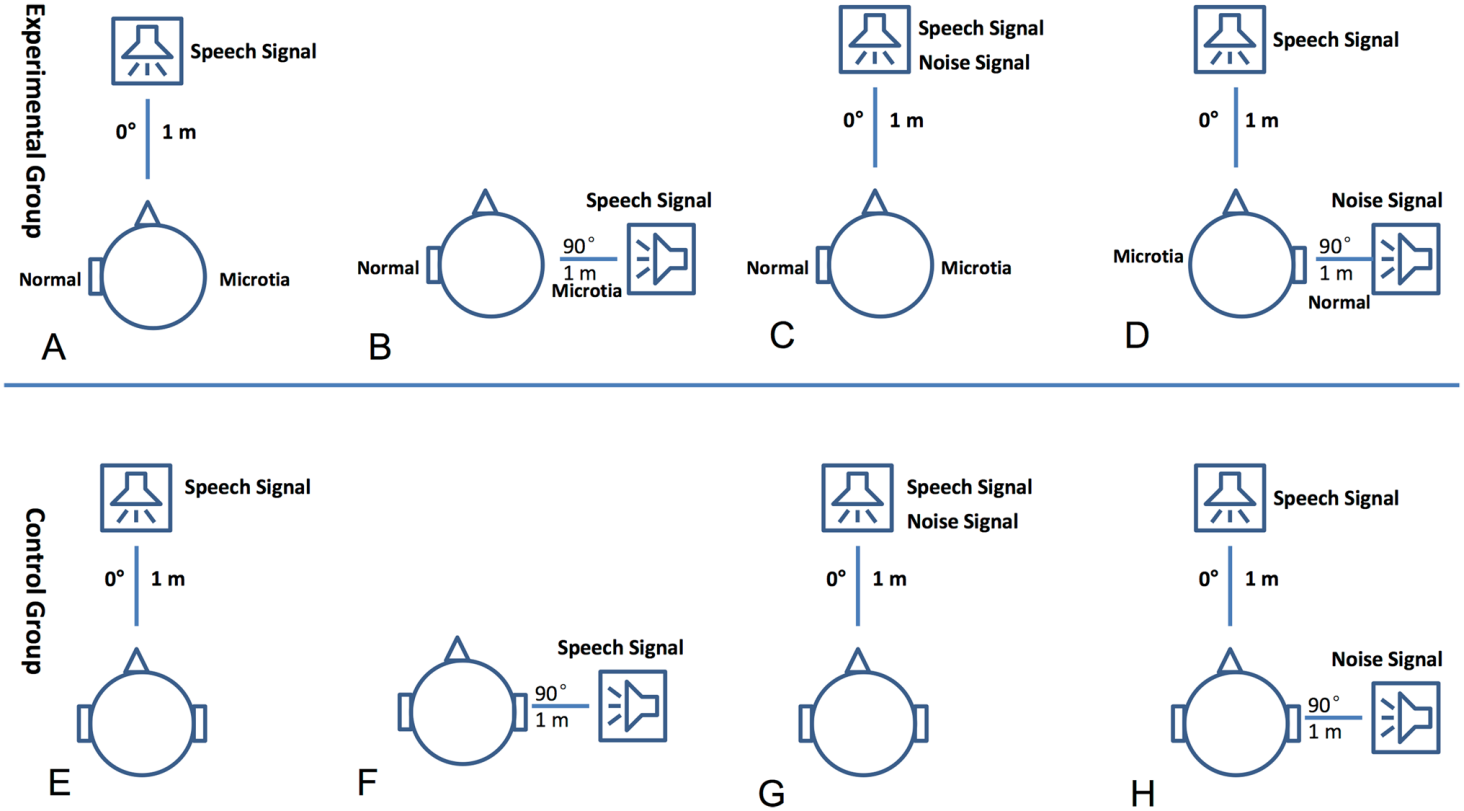
Acoustic setting for SRT test. Two speakers were used for delivering speech and noise separately. They were located 1 m away from the subject’s head and at the ear level. **A**, **B**, **C** and **D**: speaker locations for testing UMA subjects in quiet (**A** and **B**) and under masking (**C** and **D**). **E**, **F**, **G** and **H**: speaker locations for testing control subjects in quiet (**E** and **F**) and under masking (**G** and **H**)

The Speech Recognition Threshold (SRT) was assessed in a soundproof chamber using the children’s version of the Mandarin Hearing in Noise Test (MHINT), which was developed by the U.S. House Ear Institute (Wong et al., 2008). Both the speech signal and the masker (the white noise) were delivered through standard clinical audiometers and loudspeakers, placed approximately one meter from the subject’s head at ear level. When tested in quiet, the speech signal was presented at 0 azimuth under one condition (Fig. 1A–E), and at 90° azimuth under the other condition (Fig. 1F) to control subjects, or lateralized to the affected ear in subjects with UMA (Fig. 1B). When tested with masking, the speech signal was presented at 0 azimuth to subjects in both groups; whereas the noise was presented at 0° azimuth under one condition (Fig. 1C–G), and at 90° azimuth under the other condition (Fig. 1H) to control subjects, or lateralized to the unaffected ear in subjects with UMA (Fig. 1D).

Each SRT test contained 10 sentences, with 10 words in each. Each sentence was regarded as being read correctly only if every word in the sentence was correctly repeated. Each subject was tested three times, each with a different set of 10 sentences (for a total of 30 sentences) (Wong et al., 2008). The SRT in quiet was defined as the lowest sound level at which the subject correctly repeated 50% of all 30 sentences. When tested under masking, the masker was presented at a fixed level (65 dB SPL) and the speech level was adjusted to reach different signal-to-noise ratios (SNRs). The SRT under masking was defined as the lowest SNR at which the subject correctly repeated 50% of the sentences. For each measurement, SRT was calculated based on the response to the previous trial.

Subjects were also asked to complete the Speech Spatial and Qualities of Hearing Scale (SSQ) (Akeroyd et al., 2013), a self-administered questionnaire. Each item of this questionnaire asks how well a listener would do in listening situations typical of real life. Because children under eight years old may not read and comprehend the questions accurately, the parental version of the SSQ was administered. Both versions are divided into three sections: speech recognition, sound localization and hearing qualities. The score on each question ranged from 0 (not at all) to 10 (perfect). The score on each section was calculated as the average of the scores of all questions in each section.

## Analysis of MRI data

### Data acquisition

All structural and functional images were acquired on a 3.0 T MR scanner (GE, USA) with an 8-channel head coil. To minimize head movement, foam pads and earplugs were applied during scanning. The participants were instructed to hold their heads still and keep their eyes closed during the MRI scan. They were also asked to avoid falling asleep or thinking of anything during fMRI. Structural data were obtained using a 3-dimensional gradient-echo sequence (192 slices, slice thickness = 1.0 mm, TR/TE/TI (inversion time) = 6.7/2.9/450 ms, matrix = 256 × 256, field of view = 240 × 240 mm, flip angle = 7°). Functional data were obtained using an echo-planar image (EPI) sequence in 36 slices at 200 time points. The slice thickness was 4.0 mm with a gap of 1 mm. The repetition time (TR) and echo time (TE) were 2000 and 30 ms, respectively. The acquisition matrix was 64 × 64 with a flip angle of 90°. The field of view (FOV) was 200 mm × 200 mm.

### Data processing

The rs-fMRI data were preprocessed using the Data Processing & Analysis of Brain Imaging (DPABI) toolbox, available at http://rfmri.org/dpabi (Yan & Zang, 2010). The main procedures included the removal of the first 10 time points, the addition of slice-timing, the correction for head motion and spatial normalization to the Montreal Neurological Institute (MNI) template, resampling with a voxel size of 3 mm^3^, linear trend removal, regression of nuisance covariates, and band-pass filtering. The data from participants (including 4 UMA patients and 3 controls) who moved their heads more than 1.5 mm in translation or 1.5 degrees in rotation were excluded.

### Network construction and analysis

Network construction and analysis were performed using the GRETNA Toolbox (http://www.nitrc.org/projects/gretna/) (Wang et al., 2015). The workflow for network construction was similar to that reported previously (Zhang et al., 2018a). To define the nodes for the brain network, the raw MRI data were divided into 90 cortical and subcortical regions of interest (ROI), with each representing a node of the network, using an atlas of Automated Anatomical Labeling (http://www.cyceron.fr/index.php/en/plateforme-en/freeware). To define the edges of the network, Pearson correlation coefficients were calculated between the regional mean time series for each pair of the 90 brain regions. The mean time series for each brain region was first acquired by averaging the time series of all voxels within that region. Therefore, a 90*90 symmetric matrix of correlations was obtained for each subject.

The global properties of small-world networks were characterized by measuring parameters such as clustering coefficient (C_p_) (Kaiser, 2008), characteristic path length (L_p_) (Schreiber, 2013), global efficiency (E_g_) (Bassett & Gazzaniga, 2011; Doron et al., 2012; Liao et al., 2017), local efficiency (E_loc_) (Achard & Bullmore, 2007; Rubinov & Sporns, 2010) and small-world parameters (λ, γ and σ) (Rubinov & Sporns, 2010). Further, the sparsity of the network was calculated as the number of existing edges divided by the number of maximum possible edges. This indirect parameter minimizes the effects of possible differences in overall correlation strength between groups. Finally, correlation matrices were computed to show changes in properties as a function of sparsity over a wide range (between 0.05 and 0.50) with intervals of 0.05.

C_p_, the clustering coefficient of nodes, is a measure of local information transmission ability in networks and is calculated as the ratio of the actual number of edges connected to the node $(\mathrm{Ei})$ and the maximum number of possible edges of the node $(\mathrm{Ki}\left(\mathrm{Ki}-1\right)/2)$:

$$\frac{2\mathrm{Ei}}{\mathrm{Ki}\left(\mathrm{Ki}-1\right)}$$

L_p_, the minimum length between two nodes in a network, is calculated as:

$\mathrm{L}=\frac{1}{\mathrm{N}\left(\mathrm{N}-1\right)}\sum_{\mathrm{i},\mathrm{j}\in \mathrm{V},\mathrm{i}\ne \mathrm{j}}{\mathrm{l}}_{\mathrm{ij}}$(l_ij_: the minimum length between node i and node j. N: the number of node in the network)E_global_ is defined as global information transmission ability in networks and is calculated as:

$${\mathrm{E}}_{\mathrm{global}}=\frac{1}{\mathrm{N}\left(\mathrm{N}-1\right)}\sum_{\mathrm{i},\mathrm{j}\in \mathrm{V},\mathrm{i}\ne \mathrm{j}}{1/\mathrm{l}}_{\mathrm{ij}}$$

Elocal is defined as local information transmission ability in networks and is calculated as:

$${\mathrm{E}}_{\mathrm{local}}=\frac{1}{\mathrm{N}}\sum_{\mathrm{i}\in \mathrm{V}}\mathrm{E}\left(\mathrm{i}\right)$$

The small-world network parameter γ is the ratio of clustering coefficients in real and random networks, with Cp and Cr denoting the average clustering coefficients of research and random networks, respectively. The small-world network parameter λ is the ratio of path lengths in real and random networks, with Lp and Lr representing the average path lengths of research and random networks, respectively. These two parameters reflect changes in real brain networks in relation to a random or regular network. In addition, σ, the scalar measurement of a small-world network, was determined. The small-world parameters γ, λ, and σ were calculated as:

$$\upgamma\frac{\mathrm{Cp}}{\mathrm{Cr}} =\uplambda \frac{\mathrm{Lp}}{\mathrm{Lr}}=\upsigma =\frac{\upgamma }{\uplambda }$$

The nodal properties of the brain network were examined by measuring three parameters: nodal degree (NDi), representing the number of links connected to a node; nodal efficiency (NEi), representing the efficiency of parallel information transfer to a node; and nodal betweenness (NBi), representing the efficiency of information flow between one particular node and all other nodes (Rubinov & Sporns, 2010). The nodal properties NDi, NEi and NBi were calculated as:

$$\mathrm{NDi}=\sum_{\mathrm{j}\in \mathrm{N}}{\mathrm{a}}_{\mathrm{ij}}$$

$$\mathrm{NEi}\hspace{0.17em}=\frac{1}{\mathrm{N}}\sum_{\mathrm{i}\in \mathrm{N}}\mathrm{Enode},\mathrm{i}$$

$$\mathrm{NBi}=\frac{1}{\left(\mathrm{N}-1\right)\left(\mathrm{N}-2\right)}\sum_{\begin{array}{c}h,j\in N\\ h\ne j,h\ne i,j\ne i\end{array}}\frac{{\mathrm{P}}_{\mathrm{hj}}\left(\mathrm{i}\right)}{{\mathrm{P}}_{\mathrm{hj}}}$$

In addition, the parameter network hubs of the brain functional networks corresponding to these two groups of participants were calculated. Hubs are highly connected nodes in networks, with greater degrees than the average network degree. Specifically, the metrics (NDi, NEi and NBi) of the hub were at least one standard deviation greater than the mean network node metrics. Hubs are thought to play a vital role in brain functional networks, facilitating efficient communication and resilience to injury across the network.

### Statistical analysis

All data in this paper are presented as mean ± SD. The demographic and clinical data were analyzed by Fisher’s exact tests and two-sample t-tests, as appropriate. All statistical analyses were performed using SPSS 21.0 software, with P < 0.05 considered statistically significant. Differences between the UMA and control groups in global and nodal properties were determined by nonparametric permutation tests with 10,000 repetitions. The area under the curve of each network metric was calculated, with global and regional parameters in the two groups compared by two sample t-tests. Before the permutation tests, multiple linear regression analyses were performed, with age, gender and level of education as covariates. The Bonferroni method with corrected p < 0.05 was used to correct for multiple comparisons. The results of two-sample test were visualized the Brainnet Viewer (http://www.nitrc.org/projects/bnv/).

## Results

### Demographic characteristics and the results of cognitive tests

Analysis of the clinical and demographic characteristics of the two groups showed no significant differences (P > 0.05) in gender distribution, age and level of education. The mean PTA threshold of the affected ear in the UMA group was significantly higher than the mean PTA threshold of the matched ear in the control group. These UMA children have normal bone conduction thresholds. Although the difference was not statistically significant, the mean PTA was roughly 3 dB higher in the unaffected ears of the UMA group than in matched ears of the control group. There were no significant between-group differences in the scores of the MoCA and WISC-IV tests (Table 1).

**Table 1:** Demographic characteristics and cognitive test results

	UMA(n = 40)	NC(n = 40)	P value
Age(year)	9.00 ± 2.74	9.05 ± 2.29	0.93^a^
Sex(male/female)	25∕15	25∕15	1^b^
Deafness side(left/right)	13∕27	NA	NA
Education level(year)	2.63 ± 1.85	2.79 ± 1.46	0.67^a^
Handness	R	R	1^a^
PTA threshold (dB HL)*	72.12 ± 4.51	9.00 ± 2.58	< 0.001
MoCA score	28.5 ± 1.4	29.0 ± 0.9	> 0.05
WISC-IV score	97.8 ± 9.3	101.6 ± 7.9	> 0.05

Data were presented as mean ± standard deviation

*UM* unilateral microtia-atresia, *NC* normal control, *NA* not applicable, *MoCA* Montreal Cognitive Assessment, *WISC *Wechsler Intelligence Scale for Children

*PTA threshold was tested from the affected ear in the UM group and averaged from the matched ears in the control

^a^Two-sample t-tests

^b^Fisher’s exact test

### Speech recognition

When tested in quiet at 0° azimuth (Fig. 1A–E), the mean SRT was significantly higher in the UMA than in the control group (26.44 ± 1.33 dB SPL vs. 20.78 ± 2.33 dB SPL, t = 13.34; p < 0.001). This difference was ~ 3 dB larger than the difference in PTA between the unaffected ears of the UMA group and the matched ears of the control group. The difference was even greater when the signal was delivered at 90° azimuth (Fig. 1B–F), which was lateralized to the affected ears in the UMA group (29.27 ± 2.02 dB SPL vs. 21.44 ± 3.05 dB SPL t = 13.53; p < 0.001). In the UMA group, the SRT was increased by ~ 3 dB when the sound source was moved from 0 to 90 degrees, suggesting an increase in the shadow effect when the sound source was lateralized.

In the masked SRT, when both the speech and the masker were delivered at 0° azimuth (Fig. 1C–G), the SRT (expressed as SNR) was 1.24 ± 0.54 dB in the UMA group, significantly higher than the -1.42 ± 1.02 dB in the control group (t = 14.57; p < 0.001). Because both the masker and speech were presented at a level well above PTA, the between group difference was not due to the difference in PTA between the unaffected ears of subjects with UMA and matched control ears. When speech was delivered at 0° and the masker at 90° or lateralized to the unaffected ear (Fig. 1D–H), the SRT was 4.08 ± 1.71 dB in the UMA group and -7.56 ± 2.06 in the control group (t = 27.49; p < 0.001).

### SSQ scores

Children with UMA performed poorer in directional discrimination than control subjects, as shown by their significantly lower spatial scores (5.98 ± 1.27 vs. 8.21 ± 0.84, t = -9.29; p < 0.001). Additionally, the speech recognition (6.86 ± 1.17 vs. 8.53 ± 0.69; t = -7.77, p < 0.001) and hearing quality (6.69 ± 1.21 vs. 8.29 ± 0.80; t = -6.99, p < 0.001) scores were significantly lower in the UMA than in the control group.

## MRI data

### Global parameters

**Fig. 2:**
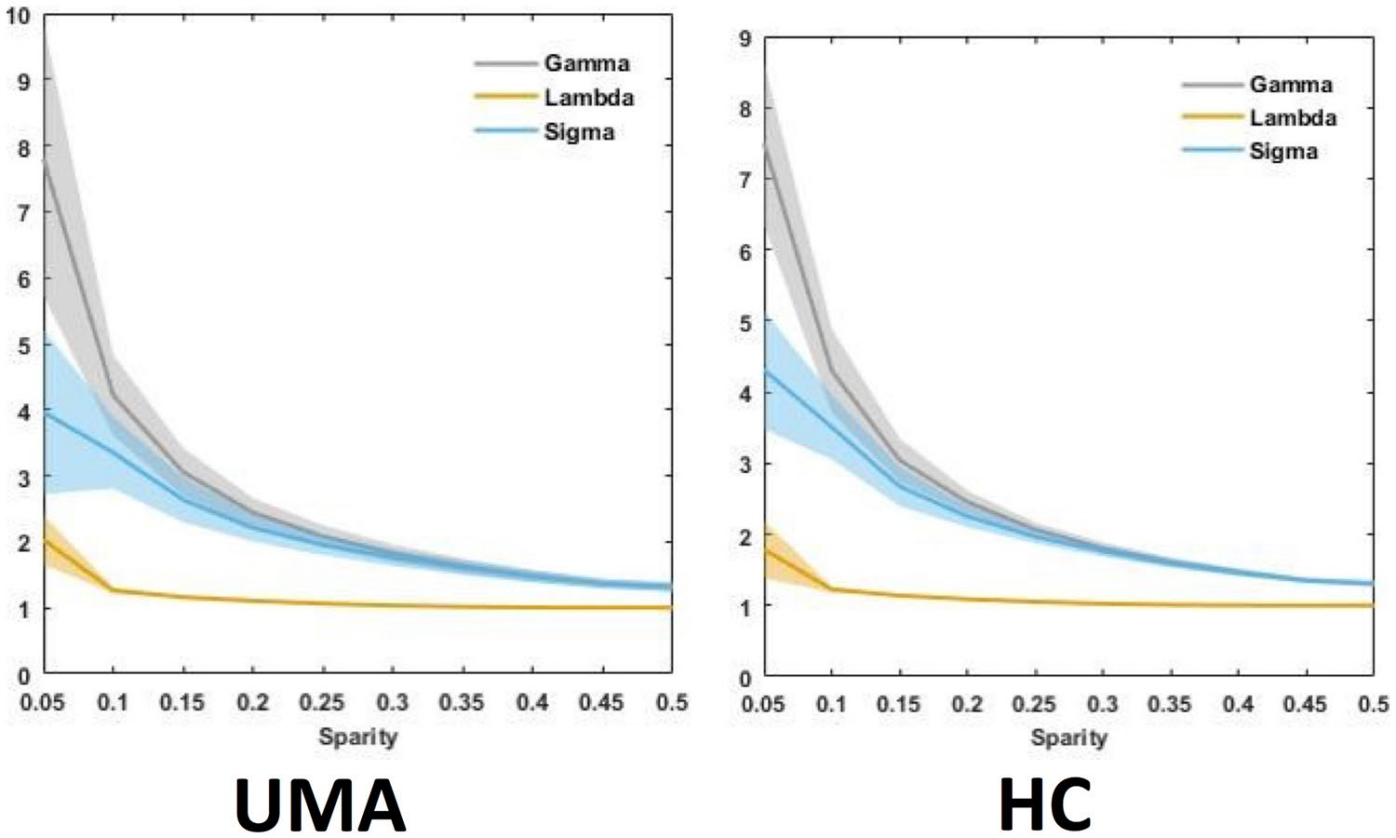
Global network parameters(γ, λ, σ) of small-world network in children with UMA and NC over the selected range of sparsity thresholds

**Fig. 3:**
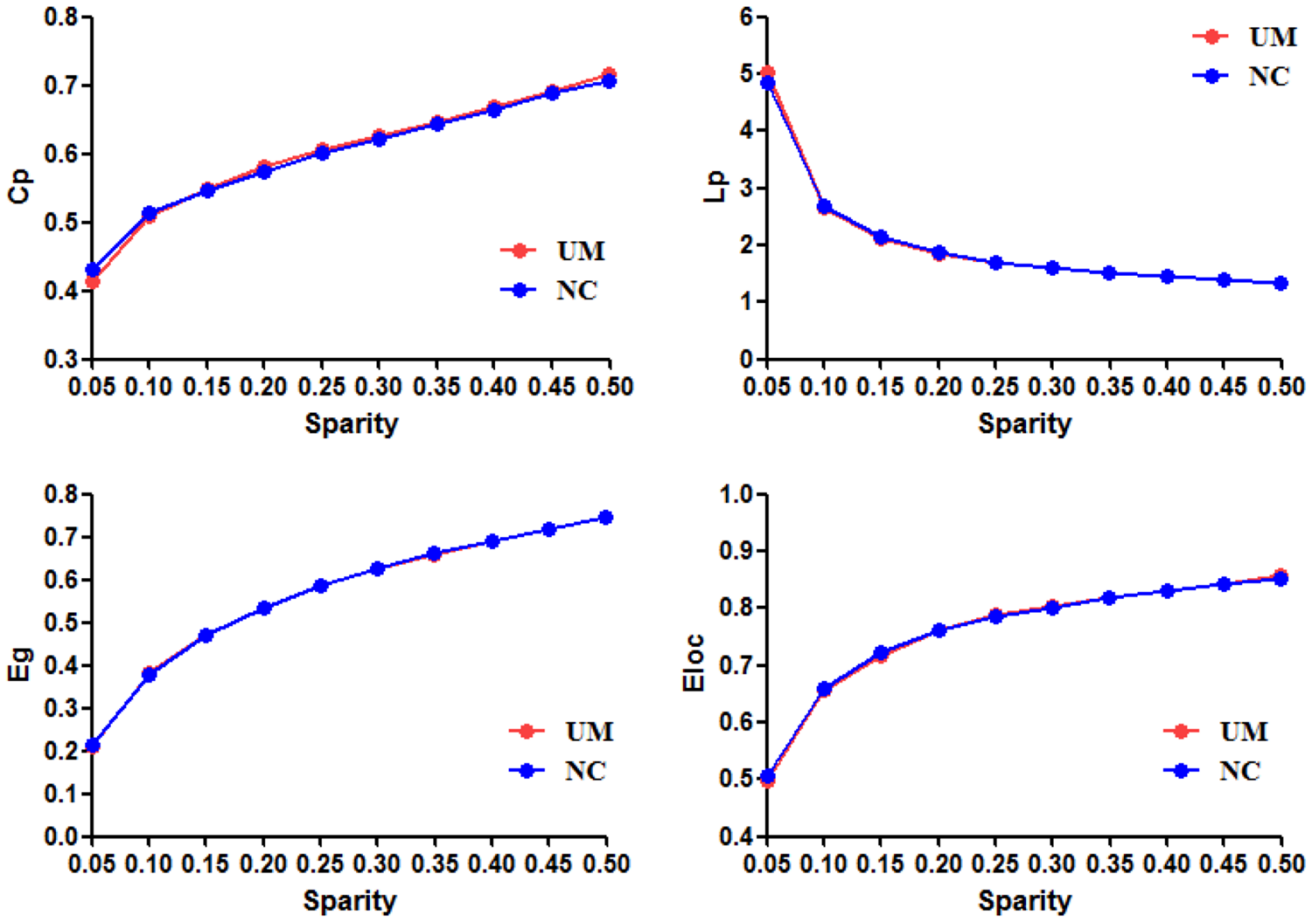
Global network parameters in children with UMA and NC over the selected range of sparsity thresholds. Bars and error bars represent mean values and standard error, respectively. Cp: clustering coefficient; Lp: characteristic path length; Eg: global efficiency; Eloc: local efficiency

Overall, there were no significant between-group differences in any of the global parameters tested in this study. Table 2 and Fig. 2 summarize the several measures of small-world networks in the two groups, including Cp, Lp, Eg and E_loc_. Figure 3 shows the changes in these four parameters as a function of sparsity in the range between 0.05 and 0.5. The curves from the two groups overlapped with each other. The two measures of small-world efficiencies were also identical in the two groups, with almost identical path lengths (λ ≈ 1) and high clustering coefficients (γ > 1).

**Table 2:** Global network metrics in UMA patients and controls

Global network measures	UMA group	Control group	t value	p value
Cp	0.272±0.010	0.273±0.017	-0.247	0.808
Lp	0.872±0.048	0.882±0.075	-0.547	0.587
γ	0.873±0.106	0.952±0.172	-1.904	0.064
λ	0.498±0.019	0.502±0.026	-0.6	0.551
σ	0.762±0.105	0.820±0.162	-1.462	0.152
Global efficiency	0.257±0.005	0.256±0.011	0.404	0.689
Local efficiency	0.344±0.006	0.346±0.008	-0.966	0.339

Data were presented as mean ± standard deviation

### Regional parameters

**Fig. 4:**
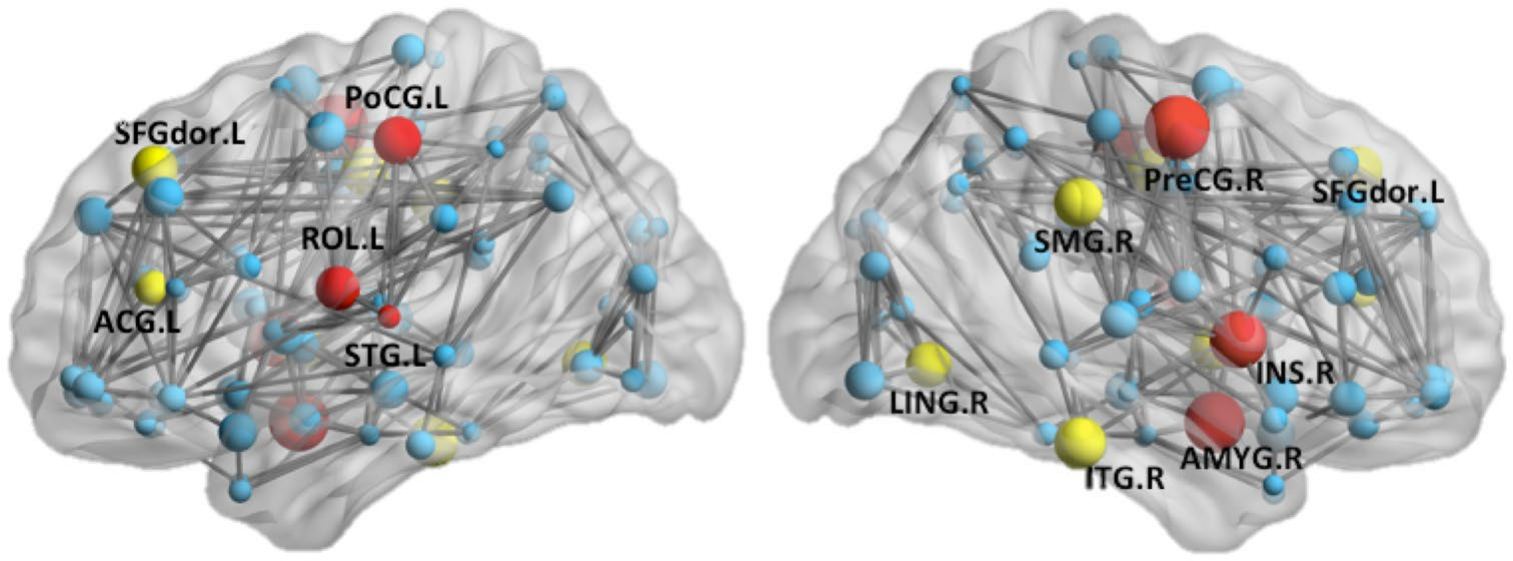
Surface visualization of brain regions showing significant between-group differences in NBi. Yellow balls: UMA > NC; Red balls: UMA < NC, blue balls: no difference. PoCG: postcentral gyrus; SFG dor: dorsolateral part of superior frontal gyrus; ROL: Rolandic operculum;ACG: anterior cingulate cortex; STG: superior temporal gyrus; PreCG: precentral gyrus; SMG: supramarginal gyrus; INS:insula; ITG: inferior temporal gyrus; LING: lingual gyrus

Among the three nodal network metrics tested, only NBi differed significantly between the UMA and control groups after Bonferroni correction. Compared with the controls, UMA patients showed larger NBi in many brain regions, including the right pallidum, right lenticular nucleus, left anterior cingulate cortex, right posterior cingulate cortex, right supramarginal gyrus, right lingual gyrus, dorsolateral part of left superior frontal gyrus, and right inferior temporal gyrus (Fig. 4). Most of these regions were in the visual network and default mode network (DMN). Moreover, the involved regions were mostly lateralized to the side of deafness. Lower-than-control NBi was also seen in the UMA group, predominantly in the auditory network, including the left superior temporal gyrus, and the attention network, including the right amygdala, right precentral gyrus, left postcentral gyrus, left Rolandic operculum, and right insula.

**Fig. 5:**
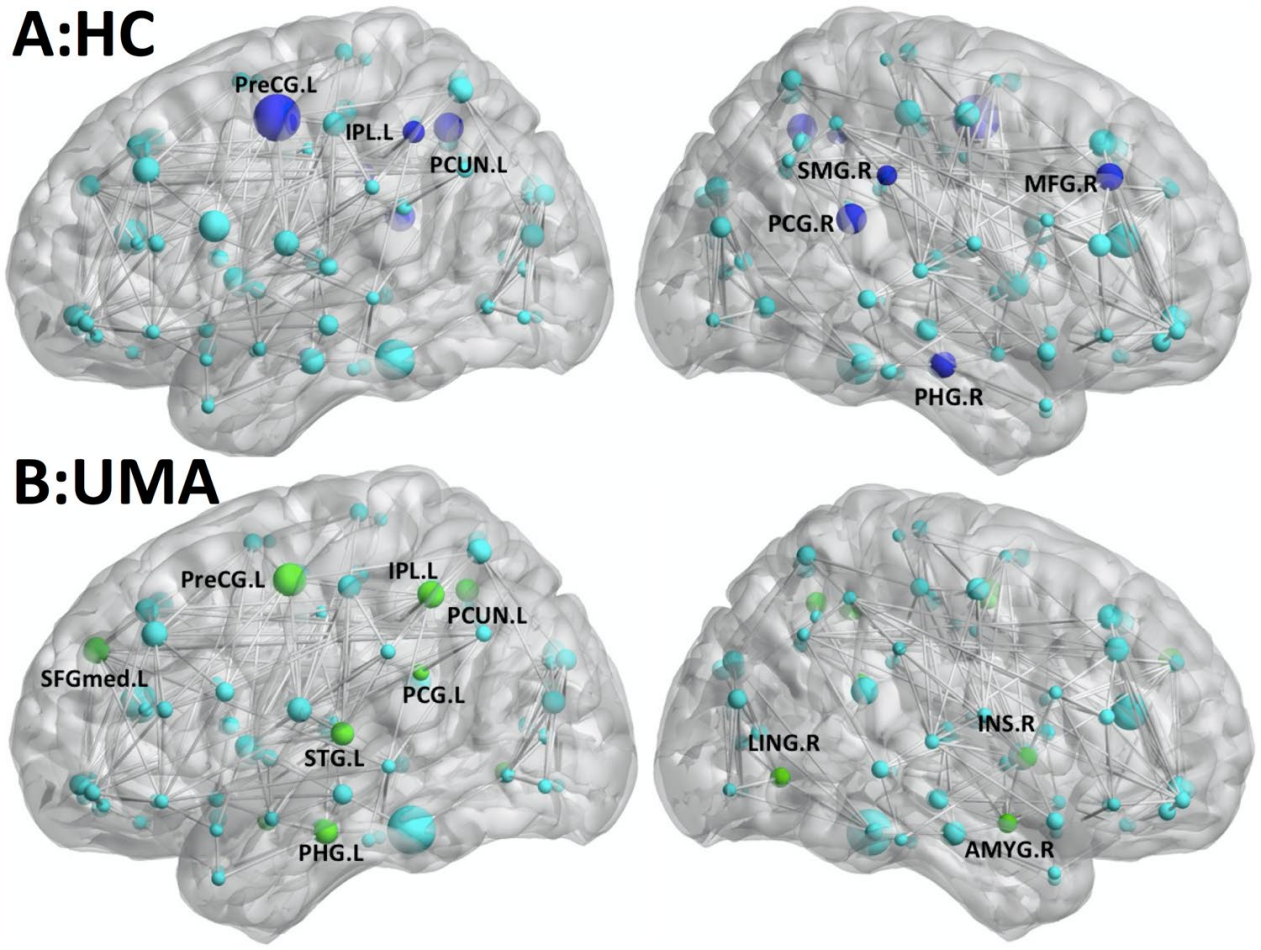
Surface-rendered plots of the functional correlation network and hubs for each hemisphere superimposed on inflated standard brains. (**A**) For HC, (**B**) for UMA patients. Gray lines indicate edges or functional connections

Figure 5 shows the hub regions of the brain functional networks for the two groups of subjects. Seven brain hub areas were identified in the healthy controls and 10 in patients with UMA.

## Discussion

To our knowledge, the present study is the first to evaluate the effects of congenital UCHL on speech perception under noise conditions, cognitive function and brain network properties in an appreciable number of subjects with UMA. These subjects showed a deficit in speech perception when tested with SRT under quiet conditions, which was exacerbated when subjects were tested under noise masking. Reduced hearing ability was also demonstrated by their poorer scores on the SSQ. Although rs-fMRI showed no between-group differences in efficiency measurements of small-world networks, UMA patients showed higher NBi in visual networks and DMN, and lower NBi in auditory and attention networks. Behavioral tests failed to show any cognitive difference between the UMA and control groups. Together, these findings demonstrated that subjects with UMA have defects in sound localization and speech perception, especially when masked by background noise. Moreover, the congenital UCHL caused by UMA resulted in brain reorganization in sensory and higher-order networks.

## Reduced hearing functions and potential mechanisms

This study found that SRT under quiet conditions was about 6 dB higher in subjects with UMA than in control subjects. This difference was due not only to the ~ 3 dB difference in PTA between unaffected ears in the UMA group and the control group. Rather, the remaining 3 dB difference was likely due to the disadvantage resulting from unilateral hearing in subjects with UMA. This disadvantage was likely due to the absence of binaural summation in these subjects, which provides a ~ 3 dB benefit near the individual threshold (Hirsh, 1948; Marks, 1987; Snik et al. 2015). The between group difference was even larger (~ 8 dB) when the sound source was lateralized to the affected ear in subjects with UMA. The ~ 3 dB increase in SRT observed in the UMA group when the speech source was moved from 0- to 90-degrees azimuth may have been due to the increased shadow effect on speech.

Performance of subjects on the masked speech recognition test was tested at 65 dB HL, and expressed as the SNR required for 50% correct responses. The two UMA and control groups showed a ~ 3 dB difference when the speech source and masker was both located at 0° azimuth, a difference similar to that observed for SRT under quiet conditions. Because these tests were performed well above the hearing threshold of the affected ear, the difference was not likely due to differences in PTA. When speech was delivered at 0° and the masker was at 90° or lateralized to the unaffected ear, the SRT (the SNR required for 50% correct perception) was increased by 3 dB in the UMA group, from 1.24 ± 0.54 dB at 0° to 4.08 ± 1.71 dB at 90°, but was reduced by 6 dB in the control group, from -1.42 ± 1.02 dB at 0° to -7.56 ± 2.06 at 90°, resulting in a between-group difference of approximately 12 dB. This increased difference was likely due to the increased masking to the unaffected ear in subjects with UMA and the reduced masking to the contralateral ear in control subjects. If the shadow effect from 0° to 90° is 3 dB, masking to the unaffected ear should also be increased by 3 dB when the masker source is lateralized to the unaffected ear in UMA subjects. The same magnitude of decrease in masking level should apply to the contralateral ear in the control subjects. Taken together, speech at 0-azimuth and a masker at 90-azimuth, lateralized to the affected ear, in subjects with UMA, provided a total 6 dB benefit to control subjects due to a change in shadow effect.

This magnitude, however, was not sufficient to account for the 12 dB difference between the groups. The difference not due to changes in the shadow effect may be due to the binaural squelching effect, defined as the difference in performance between monaural listening by the ear with better SNR and binaural hearing when speech and noise are presented on the opposite sides. The ear of the control subjects contralateral to the masker received a much higher SNR. Although speech and noise were not on opposite sides in our setting, they were largely separated and should therefore constitute binaural squelch, which takes advantages of differences between competing signals to both ears (e.g., in time/phase, level, and spectrum) (Carhart, 1965a, b). This effect is thought to result from nuclei in the brainstem processing binaural differences in signals. During this process, speech and noise are integrated and then separated into auditory objects, because the interaural differences in speech and noise differ, with the central nervous system using these differences to suppress noise (Bernstein et al., 2017; Cox & Bisset, 1984; Dincer D'Alessandro et al., 2015; Sargent et al., 2001; Snik et al., 2015).

The poorer speech recognition ability of subjects with UMA was consistent with their lower scores on the SSQ, a subjective questionnaire frequently used to evaluate subjects with UHL (Banh et al., 2012; Gatehouse & Noble, 2004; Zhang et al., 2015). The speech and quality domains of the SSQ contain complex listening recognition and discrimination situations that are frequently encountered in real life. In the present study, spatial hearing was the domain showing the largest gap between the UMA and control groups. This was expected, as the binaural cues for sound localization in azimuth are virtually unavailable or ineffective in UMA subjects with severe-to-profound UHL.

## Impact of congenital UCHL on brain networks

Advances in rs-fMRI have enabled the objective exploration of the intrinsic functional organization of the human brain and how it is changed by diseases (Dosenbach et al., 2007). Although rs-fMRI scanning data are easy to obtain, data analysis is challenging and involves complicated theories. A small-world network or small-worldness refers to a network in which most nodes (individual subjects in a society or individual nuclei in the brain) can be reached by a small number of hops or steps. Therefore, strangers can be linked by a short chain of acquaintances, an idea first mentioned in a short story published in 1929 by the Hungarian writer Frigyes Karinthy. Specifically, a small-world network is characterized as having a typical distance between two randomly selected nodes that increases in proportion to the logarithm of the number of nodes in that network (Mathias & Gopal, 2001; Pandit & Amritkar, 1999; Watts & Strogatz, 1998; Yang & Holland, 2005). The human brain appears to be an interconnected system with the properties of a small-world network, an architectural feature that facilitates efficient information segregation and integration at low cost (Liao et al., 2017). Moreover, this organization is dynamic and can be altered by changes in input from sense organs (Zhang et al. 2018a) and by development (Cao et al., 2016; Chen et al., 2013; Duan et al., 2014; Lee et al., 2019), aging (Micheloyannis et al. 2009; Wu et al. 2013), and neurological disorders (Batalle et al., 2012; Li et al., 2019; Ponten et al., 2007; Rowland et al., 2018; Tijms et al., 2015; Zeng et al., 2017).

The present study evaluated quantitative properties of small-world networks, including their global properties (Cp, Lp, Eg and Eloc) and small-world parameters (λ, γ and σ). There were no significant differences in global measures of small-world properties between the UMA and control groups, indicating that these connective properties in human brains were not significantly altered by long-term unilateral deprivation of auditory input, starting before the critical period of brain development. These findings were consistent with the results of rs-fMRI in patients with long-term sensorineural UHL established during adulthood (Zhang et al., 2018a). Testing of subjects with USHL several days after the onset of the hearing loss showed a significant increase in clustering coefficient and a decrease in characteristic path length (Xu et al., 2016). Discrepancies across studies may have been due to the reversal of initial changes by later adaptive and compensatory functional reorganizations (Zhang et al., 2018a). This may also be applicable to UCHL in subjects with UMA.

Although significant global topological differences were not detected, alterations in nodal properties were observed in the sensory network as well as in higher-order cognitive networks. NBi is a regional index that quantifies the number of short paths that pass through a specific node divided by the total number of short paths in the entire network. Thus, NBi reflects the degree of information flow of any particular brain region in the entire network. High NBi, for example, indicates highly traveled paths within a region. The present study found NBi was reduced in auditory regions, such as the superior temporal gyrus, and increased in visual regions, such as the lingual gyrus. These changes suggest cross-modal differences in brain organization in subjects with UMA. Partial deprivation in one sensory modality has been shown to affect the functions of other sensory modalities that remain intact. For example, rs-fMRI scanning in patients with UHL found that auditory sensory deprivation affected the function of the visual brain (Liu et al., 2015; Wang et al., 2014; Yang et al., 2014; Zhang et al., 2018a, b) and that NBi was decreased in the bilateral Heschl’s gyrus and the bilateral superior and middle temporal gyri of the auditory network (Zhang et al., 2018a). In the present study, however, this decrease was observed only in the left superior temporal gyrus. This discrepancy may be due to the inclusion in our study of subjects with right-sided UMA alone, whereas the other studies assessed subjects with both left- and right-sided UHL.

Language tests showed that activation of primary visual processing regions was significantly greater, whereas activation of secondary visual centers was significantly lower, in children with USHL than in controls (Schmithorst et al., 2014). Interestingly, the present study also found that activation in the auditory brain was significantly greater in subjects with USHL than in controls when performing these tests. Increased activation in the auditory brain may reflect the increased effort in auditory processing due to auditory deprivation and/or stronger feedback to the primary auditory center associated with the enhanced visual process (Schmithorst et al., 2014). In contrast, reduced activation in the secondary visual processing centers may have been due to auditory deprivation induced changes in connections between auditory and visual regions (Schmithorst et al., 2014). The increased brain activation in subjects with USHL may be counter to the changes in NBi in subjects with UMA tested in the resting state (Zhang et al., 2018a).

The present study found between-group differences in NBi in several regions of the DMN, consistent with previous findings (Wang et al., 2014; Zhang et al., 2016, 2018a; Zhang et al., 2015). The DMN is involved in cognitive processing, including in emotional processing, self-referential mental activity, conflict monitoring, memory retrieval and cognitive control (Brewer et al., 2011). The nodal alteration in the DMN observed in the present study indicates that the partial hearing loss in subjects with UMA may affect the cognitive brain. However, the scores on the MoCA and WISC tests did not differ significantly in the UMA and control groups, suggesting that rs-fMRI measures may be more sensitive than neuropsychological tests in evaluating cognitive changes in subjects with UMA. Alternatively, cognitive deficits in UMA subjects may alter cognitive performance in the classroom and speech and language acquisition that are not targeted by the MoCA and WISC tests. In addition, we found that the functional hubs in normal control group were located primarily in several DMN brain regions, which was consistent with previous functional connectome studies (Buckner et al., 2009; Dai et al., 2015; Xu et al., 2016). And similar hub distribution was also found in UMA group, which suggests the crucial roles of these hubs for relative preservation. Meanwhile, the UMA patients showed a decreased nodal strength in some hub regions, which suggests that these hubs might be preferentially targeted by congenital UHL. The increased hub brain regions in UMA group might represent a compensatory coordination for information processing.

A previous fMRI study in subjects with UHL also reported abnormal reorganization in the attention network (Zhang et al., 2018a, b), which is thought to be responsible for attentional reorientation in response to salient relevant external stimuli or internal goals (Majerus et al., 2018). Listeners with normal hearing can filter out competing sound sources, select a desired source, and quickly switch their attention among different sources using an attention network. In this study, NBi was reduced in the insula and the fronto-insular cortex of the attention network. The insula plays vital roles in detecting and orienting in response to salient external stimuli and internal events associated with auditory attention and memory (Huang et al., 2013). The fronto-insular cortex may provide executive control over switching attention in complicated conditions. In such tasks, binaural cues are critical. Sound localization and speech perception in background noise were poorer in children with UMA than in controls, suggesting that unilateral acoustic deprivation may have reduced the salience of auditory cues used to select a desired sound source, including its location, pitch, and timbre. This deprivation reduces the local information processing capacity of auditory and attention networks. Interestingly, UHL can disrupt neural tuning to localize sound in the rat primary auditory cortex (Wang et al., 2019). This may partly explain the finding that, despite improvements on sound localization tests, the localization abilities of children fitted with bone conductive devices for congenital conductive UHL were lower than the localization abilities of children with normal hearing (Nelissen et al., 2016).

## Limitations

There are still several limitations in the present study. First, the sample size is small. We only recruited 27 patients with right-sided UCM, and 4 subjects were excluded for excessive head movement. More subjects including right-sided and left-sided UCM will be scanned in further studies. And the difference between them will be researched. Second, although earplugs were used to reduce the scanner noise, it should be noted that the brain activity and intrinsic brain networks such as the auditory network and DMN can still be affected by the technological issue. Finally, as a pilot study, the results of UCM were compared with previous studies on unilateral sensorineural deafness, further research is required to make comparisons directly.

## Conclusion

In conclusion, children with UMA showed poorer performance on speech recognition and sound localization tasks compared with age- and sex-matched normal hearing controls due to the loss of binaural benefits. Subjects with UMA also showed connectome-level alterations belonging to multiple large-scale networks involved in sensory and higher-level cognitive functions. These findings provide new insights into the effects of UHL on brain development in children with UMA and suggest the need for early hearing intervention in such children.
